# Evolving Paradigms in Sepsis Management: A Narrative Review

**DOI:** 10.3390/cells13141172

**Published:** 2024-07-09

**Authors:** Min-Ji Kim, Eun-Joo Choi, Eun-Jung Choi

**Affiliations:** 1 Department of Internal Medicine, School of Medicine, Kyungpook National University, Kyungpook National University Chilgok Hospital, Daegu 41404, Republic of Korea; kmj_endo@knuh.kr; 2 Department of Anesthesiology and Pain Medicine, School of Medicine, Daegu Catholic University, Daegu 42472, Republic of Korea; sthgood9@naver.com; 3 Department of Anatomy, School of Medicine, Daegu Catholic University, Duryugongwon-ro 17gil, Nam-gu, Daegu 42472, Republic of Korea

**Keywords:** sepsis, hyperinflammation, immune paralysis

## Abstract

Sepsis, a condition characterized by life-threatening organ dysfunction due to a dysregulated host response to infection, significantly impacts global health, with mortality rates varying widely across regions. Traditional therapeutic strategies that target hyperinflammation and immunosuppression have largely failed to improve outcomes, underscoring the need for innovative approaches. This review examines the development of therapeutic agents for sepsis, with a focus on clinical trials addressing hyperinflammation and immunosuppression. It highlights the frequent failures of these trials, explores the underlying reasons, and outlines current research efforts aimed at bridging the gap between theoretical advancements and clinical applications. Although personalized medicine and phenotypic categorization present promising directions, this review emphasizes the importance of understanding the complex pathogenesis of sepsis and developing targeted, effective therapies to enhance patient outcomes. By addressing the multifaceted nature of sepsis, future research can pave the way for more precise and individualized treatment strategies, ultimately improving the management and prognosis of sepsis patients.

## 1. Introduction

Sepsis is defined as life-threatening organ dysfunction caused by a dysregulated host response to infection [1]. Alarmingly, it accounts for nearly 19.7% of all global deaths and thus severely impacts health outcomes [2,3]. Mortality rates for sepsis vary widely, from 15% to higher than 50%, depending on the region and healthcare system. For example, the MOSAICS study reported a hospital mortality rate of 44.5% for severe sepsis in Asian countries, compared with 28.6% in the U.S. and 18.4% in Australia and New Zealand [4]. These statistics underscore the urgent need for innovative treatment approaches, especially given the lack of any FDA-approved treatments for sepsis [5].

Historically, the development of treatments for sepsis was based on the classical view of its pathogenesis, focusing on the systemic inflammatory response to infection causing widespread tissue damage and organ failure. Despite great research efforts aimed at mitigating this exaggerated immune response, the outcomes have been largely unsatisfactory [6,7]. However, recent shifts in understanding sepsis have led to new research avenues, challenging the classical view by suggesting that alternative mechanisms and pathways participate in its development. Studies now focus on modulating the immune system rather than suppressing it, and recognize sepsis as a heterogeneous syndrome, which has led to more personalized treatment approaches [5,8].

This review delineates the development of therapeutic agents targeting hyperinflammation and immunosuppression in sepsis, highlighting the range of clinical trials conducted thus far. Additionally, it discusses the predominance of failures in these trials, investigates the reasons behind these failures, and outlines the current research focus aiming to bridge the gaps between theoretical advancements and clinical applications.

## 2. Results

### 2.1. Hyperinflammation Matters

#### 2.1.1. Pathogenesis of Sepsis According to the Classical View

When bacteria or viruses invade the human body, Toll-like receptors (TLRs) on sentinel cells act as an alarm system, swiftly recognizing pathogen-associated molecular patterns (PAMPs) and triggering the body’s initial line of defense [9,10]. The immune system comprises key components known as pathogen recognition receptors (PRRs), which include TLRs, RIG-I-like receptors (RLRs), nucleotide-binding oligomerization domain-like receptors (NLRs), absent in melanoma 2-like receptors (ALRs), C-type lectin receptors, and sensors for internal DNA and RNA [11]. The recognition of bacteria and viruses by PRRs is significantly influenced by the localization and structure of these receptors [11]. For instance, TLRs are categorized based on their cellular localization, which determines the types of ligands they recognize and their recognition mechanisms [12]. Bacteria are typically recognized by surface-expressed TLRs such as TLR2, TLR4, and TLR5 on innate immune cells, while viruses, due to their nucleic acid-based structures, are often identified by endosome-located TLRs, including TLR3, TLR7, and TLR9 [13,14,15,16,17]. The binding of ligands to these TLRs triggers the recruitment of adaptor molecules such as MyD88 and TRIF, initiating signaling pathways that result in the transcription and secretion of key pro-inflammatory cytokines like tumor necrosis factor-alpha (TNF-α), interleukin-1 beta (IL-1β), and interleukin-6 (IL-6), which are essential for coordinating the immune response and enhancing the body’s defense against pathogens [18].

**Figure 1:**
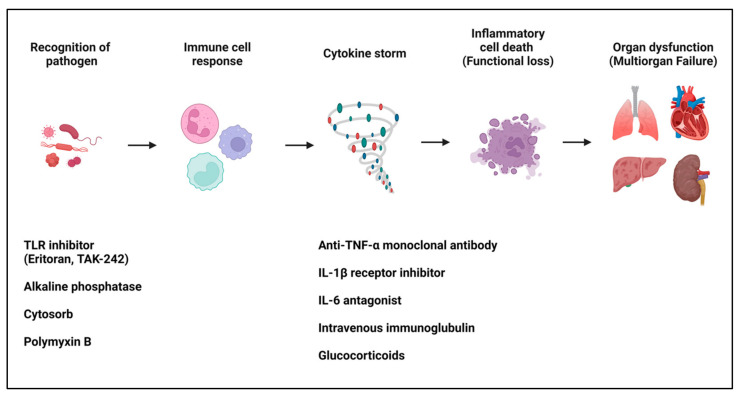
Simplified diagram explaining the pathogenesis of sepsis from a classical perspective and treatment strategies. This diagram depicts the classical pathogenesis of sepsis, underscoring the activation of the innate immune system by PAMPs via TLRs. It delineates how this activation can escalate into cytokine storms, causing systemic inflammation and multiorgan failure. Various therapeutic strategies based on this classical understanding are illustrated below, including TLR inhibitors, alkaline phosphatase, CytoSorb, polymyxin B, anti-TNF-α monoclonal antibodies, IL-1β receptor inhibitors, IL-6 antagonists, and glucocorticoids. Each treatment is positioned underneath the process it targets.

While the response of the innate immune system to PAMPs is crucial for combating infections, an excessive response can lead to a cytokine storm [19,20,21]. The pathogenesis of a cytokine storm involves excessive cytokine production, leading to systemic inflammation and extensive tissue damage, which undermines cellular and tissue integrity, impairs the function of vital organs, and ultimately results in multiorgan failure, a hallmark of sepsis [21,22]. The pathogenesis of sepsis also involves hypoperfusion or hypotension, conditions characterized by significantly reduced blood flow to tissues and markedly decreased blood pressure, respectively [22]. These conditions compound the effects of a cytokine storm by further decreasing the delivery of oxygen and nutrients to tissues, exacerbating tissue damage, and contributing to the failure of multiple organs [20]. This classical view of the pathogenesis of sepsis highlights the critical impact of an overactive immune response in severe infections (Figure 1).

#### 2.1.2. Strategies for Inhibiting Pathogen Recognition and Targeting Hyperinflammation in Sepsis

Addressing the root causes of sepsis by removing initiating factors and controlling hyperinflammation is a compelling treatment strategy. This approach, focusing on clearing pathogens, inhibiting pathogen recognition, and directly targeting pro-inflammatory cytokines or their receptors, aims to fundamentally alter the course of sepsis. By preventing the overactivation of the immune system, this strategy seeks to avert a cytokine storm and subsequent multiorgan failure, and has attracted significant interest. This logic has garnered significant interest among researchers and pharmaceutical companies, leading to the initiation of various clinical trials [23].

- Inhibiting Pathogen Recognition in Sepsis Management

In the context of targeting hyperinflammation in sepsis, the strategy of inhibiting the recognition of PAMPs using TLR inhibitors is a notable approach [24,25,26,27]. TLR4, a receptor that mediates endotoxic shock and cytokine storms associated with sepsis, has been a particular focus of interest. Eritoran, a synthetic lipid A analog, binds to the TLR4-MD-2 complex, effectively blocking the interaction between lipopolysaccharide (LPS) and TLR4 and thus inhibiting the pro-inflammatory signaling pathway [28]. This mechanism demonstrated the efficient blockade of LPS-induced cytokine production both in vitro and in animal models [28]. However, despite promising results in phase I/II clinical trials in terms of safety and tolerability, Eritoran failed to demonstrate efficacy in the ACCESS phase III randomized trial, which involved 1961 patients with severe sepsis. This led to the discontinuation of its clinical development in 2011 [29].

TAK-242 is a molecular inhibitor of TLR4 that was developed to treat sepsis by inhibiting TLR4 signaling, thereby suppressing the excessive immune responses [30]. Despite showing efficacy in in vitro and animal models by reducing fibrosis and inflammatory responses, TAK-242 failed to meet its primary endpoint of reducing mortality in patients with severe sepsis during phase III clinical trials conducted in 2013 [30,31].

- Pathogen Clearance in Sepsis Management

In addition to inhibiting the recognition of PAMPs using TLR inhibitors, another pivotal approach to mitigate hyperinflammation in sepsis involves directly neutralizing pathogen-derived components. This strategy targets the removal of elements that drive the inflammatory response, thereby reducing sepsis-induced hyperinflammation. Devices such as CytoSorb and therapeutic strategies like alkaline phosphatase (AP) administration and polymyxin B hemoperfusion exemplify this approach and aim to lessen the burden of systemic inflammation by directly eliminating cytokines, toxins, and endotoxins from the bloodstream. While the inhibition of TLR-mediated recognition targets the upstream initiation of the inflammatory cascade, pathogen clearance addresses the downstream effects, providing a broad spectrum of interventions in the complex pathophysiology of sepsis.

AP is an enzyme recognized for its role in detoxifying LPS and endotoxins produced by Gram-negative bacteria, especially in the context of sepsis [32]. Its capability to dephosphorylate and neutralize LPS can significantly mitigate inflammation and the systemic response to infection [32]. Phase II clinical trials yielded promising results, with AP improving kidney function in sepsis patients, suggesting its potential as a therapeutic agent for sepsis-associated acute kidney injury (AKI) [33]. However, despite the initial promise, the subsequent REVIVAL phase III trial did not demonstrate a significant reduction in mortality rates among patients with sepsis-associated AKI using AP [34].

Polymyxin B hemoperfusion aims to treat sepsis by focusing on the clearance of circulating endotoxins, primarily those released by Gram-negative bacteria [35]. The EUPHRATES trial, a pivotal study in this area, evaluated the efficacy of polymyxin B hemoperfusion in reducing mortality among septic shock patients with high endotoxin levels. Conducted across 55 tertiary hospitals in North America from September 2010 to June 2016, this multicenter, randomized, blinded, sham-controlled study enrolled 450 critically ill adult patients [36]. Despite the theoretical benefit of lowering endotoxin levels to improve outcomes in sepsis patients, the EUPHRATES trial concluded that polymyxin B hemoperfusion, when added to conventional therapy, did not significantly reduce 28-day mortality rates compared with the sham treatment plus conventional therapy [36].

- Strategies for Inhibiting Pro-inflammatory Cytokines or Receptor Activation

Until now, strategies have primarily focused on preventing pathogen recognition and blocking pathogen invasion or TLR signaling; however, the discussion is now shifting toward treating sepsis through immunosuppression by inhibiting the cytokines produced host–pathogen recognition. In this section, we will briefly explain the significance of each cytokine in the pathogenesis of sepsis and describe how strategies to inhibit these cytokines have been investigated, using representative clinical trials as examples.

TNF-α is one of the earliest and most extensively studied cytokines in the context of sepsis [37,38]. In murine models of sepsis, TNF-α levels sharply rise and peak remarkably early, within just 1–3 h, after LPS injection [39]. Elevated TNF-α serum levels have received significant attention in clinical settings due to their correlation with the severity of sepsis and increased mortality rates [40,41]. Preclinical studies utilizing anti-TNF-α antibodies demonstrated promising results in terms of reducing the mortality and ameliorating the symptoms of sepsis [42,43,44]. Encouraged by these findings, clinical trials targeting TNF-α have been conducted. However, despite initial optimism, these trials failed to conclusively demonstrate the efficacy of TNF-α inhibition in sepsis treatment [45,46,47]. The NORASEPT II study, as reported in The Lancet in 1998, and a 1995 JAMA study explored the potential of an anti-TNF-α monoclonal antibody (TNF-α MAb) for treating sepsis and septic shock, yielding critical insights into the challenges of targeting TNF-α in sepsis therapy [46,47]. Despite encompassing a large cohort across numerous hospitals in the USA and Canada, the NORASEPT II trial found no significant improvement in 28-day mortality rates for septic shock patients treated with TNF-α MAb compared with placebo [46]. Similarly, the JAMA study also failed to demonstrate a long-term mortality benefit at 28 days, despite showing a temporary reduction in mortality among septic shock patients shortly after treatment [47]. Both studies highlighted a crucial detail: while initial responses to TNF-α MAb suggested potential benefits, these did not translate into sustained survival improvements. Alongside these findings, the trial of the TNF-α mAb afelimomab, known as the RAMESES study, provides a nuanced perspective by demonstrating a modest but significant reduction in 28-day mortality in sepsis patients with IL-6 serum levels higher than 1000 pg/mL [45].

In the context of sepsis, IL-1β plays a crucial role beyond the initial cytokine response and significantly influences the complex cytokine network [37,48]. IL-1β is not only upregulated early in sepsis but also acts as a key player in triggering the release of other cytokines [37]. According to research conducted by Matsumoto et al., IL-1β is a key instigator within the cytokine network, initiating a sequence of events involving other critical cytokines such as IL-6 and IL-8 [37]. IL-1β is an independent prognostic factor in sepsis, with evidence that it is significantly upregulated in sepsis patients compared with patients with systemic inflammatory response syndrome, and its levels are even higher in septic shock cases, emphasizing its critical role in enhancing the inflammatory response and marking it as a key indicator of sepsis severity and prognosis [49,50]. Evidence supporting the efficacy of IL-1β inhibition in sepsis has predominantly been derived from NLRP3-deficient models. Lee et al. and Jin et al. demonstrated that NLRP3 inflammasome deficiency protects against microbial sepsis in mouse models by increasing lipoxin B4 synthesis and by enhancing survival, decreasing autophagy, and augmenting phagocytosis, respectively [51,52]. Despite the promising theoretical framework suggesting the potential of targeting IL-1β for sepsis treatment, clinical trials involving anakinra, an IL-1β receptor antagonist, have not demonstrated a significant improvement in patient outcomes. Randomized, double-blind, placebo-controlled trials that aimed to assess the efficacy of anakinra in sepsis syndrome and severe sepsis unfortunately failed to show a definitive improvement in survival rates for sepsis patients treated with anakinra [53,54].

While TNF-α and IL-1 drive the initial responses during the early stages of sepsis, the blood levels of IL-6 can be maintained over a longer period [37]. The persistent presence of IL-6 is inversely correlated with survival rates in patients with bacterial sepsis and septic shock, indicating that IL-6 plays a significant role in the progression and poor outcome of sepsis [55,56,57]. Evidence from preclinical studies supports the therapeutic potential of IL-6 blockade, showing that antibodies targeting IL-6 can improve survival and physiological responses in sepsis models [58,59]. However, despite these promising findings, the results are not uniformly positive. Contradictory results have emerged, such as the finding that high doses of recombinant IL-6 did not elicit adverse effects in healthy dogs, indicating that IL-6 plays a complex role in sepsis [60]. Furthermore, IL-6-knockout mice do not demonstrate enhanced survival upon the induction of sepsis by cecal ligation and puncture, adding to the debate about the dual role of IL-6 in sepsis dynamics [61]. The role of IL-6 in immunity has dual significance, not solely due to the mixed outcomes described above but because of its inherent pro-inflammatory and anti-inflammatory functions, marking it as a double-edged sword in the immune response [62,63]. This characterization stems from the unique capacity of IL-6 to both initiate pro-inflammatory reactions, potentially exacerbating conditions like sepsis, and concurrently trigger anti-inflammatory pathways, which are essential for the body’s healing and recovery processes [62]. Due to inconsistent preclinical results, few clinical studies initially targeted IL-6 in classical sepsis. However, recent findings of elevated IL-6 levels in COVID-19 patients have spurred numerous clinical trials exploring IL-6-targeted strategies [64,65,66,67]. These findings suggest that, while IL-6 antagonists do not benefit all hospitalized patients with mild-to-moderate COVID-19 symptoms, they may offer advantages in severe COVID-19 cases [67].

Intravenous immunoglobulins (IVIg), particularly those enriched with IgM (IgGAM), are being explored to manage sepsis-induced immune dysregulation [68]. IVIg aim to mitigate inflammation and tissue damage by interacting with excess cytokines and complement factors involved in cytokine storms. The clinical rationale for IVIg therapy encompasses the recognition and clearance of pathogens, the inhibition of mediator gene transcription, and the exertion of anti-apoptotic effects in immune cells [69]. Despite its theoretical benefits, clinical evidence, including guidelines of the Surviving Sepsis Campaign, points to inconsistent results with IVIg, leading to recommendations against their routine use [69]. Research studies such as SBITS and ESSICS have further demonstrated the challenges, showing no significant benefits in mortality rates among IVIg-treated patients, which underscores the need for a cautious approach to IVIg application in sepsis therapy [70,71].

Glucocorticoids have been considered as potential treatment options for sepsis due to their broad anti-inflammatory effects [72]. As pan-inflammation inhibitors, glucocorticoids exert their effects by interacting with glucocorticoid receptors, leading to the modulation of various inflammatory pathways. This interaction primarily results in the inhibition of transcription factors such as NF-κB and AP-1, which are crucial for the synthesis of pro-inflammatory cytokines like TNF-α, IL-1, and IL-6 [73]. The suppression of these cytokines is critical to manage the systemic inflammation observed in sepsis. However, the efficacy of glucocorticoid treatment in sepsis remains highly controversial, as evidenced by the divergent outcomes in major clinical trials [74,75,76,77,78,79]. A 2002 French trial and the 2018 APROCCHHS trial both reported reduced mortality in septic shock patients treated with a combination of hydrocortisone and fludrocortisone [74,76]. In contrast, the CORTICUS trial showed that, while hydrocortisone sped up septic shock reversal, it did not improve the mortality rates, and the HYPRESS trial found no significant prevention of septic shock or improvement in survival outcomes [74,78]. Further complicating the picture, the VANISH trial found that adding hydrocortisone to vasopressor treatments did not improve the mortality or affect kidney failure rates [79]. Similarly, the ADRENAL trial observed that, despite hastening septic shock resolution and reducing the need for blood transfusions, hydrocortisone did not significantly improve the 28- or 90-day mortality rates [75]. Overall, the role of glucocorticoids in sepsis remains a complex and debated topic in critical care, and further research is needed to clarify their place in sepsis management protocols.

- Strategies for Inhibiting Damage-Associated Molecular Patterns (DAMPs)

As sepsis treatment strategies focusing on cytokine inhibition encountered setbacks, researchers discovered that cytokines do not merely surge momentarily; rather, they are part of a sustained immune response in sepsis. It has now been recognized that not only is there an initial response to infections, but multiple secondary cytokine releases also occur [80,81,82]. These subsequent releases can be triggered by the destruction of cell membranes during the initial cytokine storm, which releases materials that serve as further immune triggers [82]. In this sequence of septic responses, the complement system, particularly through the activation of components such as C3a and C5a, plays a crucial role in destructive processes during sepsis [83]. Key substances include high mobility group box 1 (HMGB1) and heat shock proteins (HSPs), which are DAMPs released from cells during sepsis. Following the release of such DAMPs during sepsis, neutrophil extracellular traps (NETs), which are webs of DNA, histones, and antimicrobial proteins formed by neutrophils, also contribute to the inflammatory cascade, exacerbating the response and potentially leading to further tissue damage in the septic environment [84]. While inhibitors of HMGB1 and HSPs have demonstrated efficacy in murine experimental models, these findings have not been translated into clinical studies [39,85,86,87,88].

### 2.2. Immunosuppression Matters

#### 2.2.1. Mechanisms and Consequences of Immunosuppression in Sepsis

**Figure 2:**
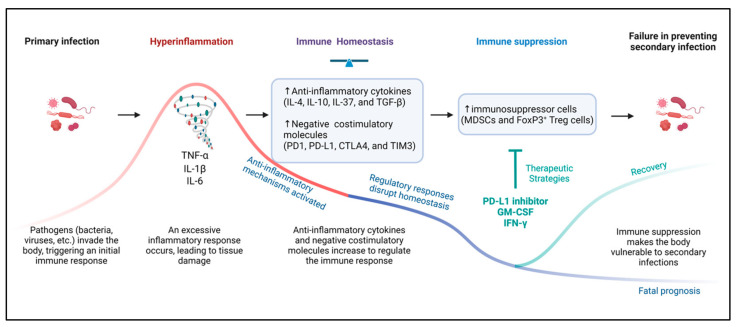
Mechanisms and consequences of immunosuppression in sepsis and potential therapeutic strategies. During hyperinflammatory states, several anti-inflammatory mechanisms are activated to mitigate inflammation and promote tissue recovery, aiming to restore immune homeostasis. These include the upregulation of negative costimulatory molecules such as PD-L1 and PD-1 on immune cells, the increased production of anti-inflammatory cytokines like IL-4, IL-10, IL-37, and TGF-β, and the expansion of the myeloid-derived suppressor cell (MDSC) population together with an increase in FoxP3^+^ T cells. These regulatory responses can disrupt immune homeostasis, leading to immunosuppression characterized by suppressed T cell responses and increased apoptosis. This immunosuppression can result in failure in preventing secondary infection. Therapeutic strategies targeting these pathways include GM-CSF to enhance neutrophil activity, anti-PD-L1 antibodies to alleviate T cell exhaustion, and IFN-γ to boost macrophage capabilities.

The classical view of sepsis pathogenesis emphasizes the need to inhibit hyperinflammation, which is thought to drive organ damage during the early stages of infection. However, an alternative concept has emerged, notably represented by the Hotchkiss group in the early 2000s, which focuses on the role of immunosuppression in the later stages of sepsis [89]. This view posits that, while hyperinflammation causes initial organ damage, the subsequent immunosuppression prevents the body from effectively combating secondary infections, leading to further deterioration in the patient’s condition. This perspective highlights the importance of understanding the dual phases of sepsis—initial hyperinflammation followed by immunosuppression—to develop more effective management strategies (Figure 2).

In hyperinflammatory states, several anti-inflammatory mechanisms are activated to mitigate inflammation and promote tissue recovery [90,91]. These include the upregulation of negative costimulatory molecules such as PD-L1 and PD-1 on immune cells, the increased production of anti-inflammatory cytokines like IL-4, IL-10, IL-37, and TGF-β, and the expansion of the myeloid-derived suppressor cell (MDSC) population together with an increase in FoxP3^+^ T cells [91,92,93,94,95,96,97]. The expansion of MDSCs and regulatory T cells plays a critical role in the suppression of T cell responses. MDSCs exert their suppressive effects by releasing arginase, nitric oxide, and reactive oxygen species (ROS), which inhibit T cell receptor signaling and induce metabolic changes that impair T cell function and promote T cell apoptosis [98]. Similarly, expanded regulatory T cells contribute to immune paralysis by secreting immunosuppressive cytokines such as IL-10 and TGF-β, which further inhibit the activation and function of effector T cells [99]. Although TGF-β is an anti-inflammatory cytokine that suppresses T-cell responses and inhibits the effector T cell function, which has detrimental effects on disease outcomes by promoting fibrosis [97].

In sepsis, similarly to its role in cancer, the interaction between PD-1 and PD-L1 impairs T cell function by inhibiting cytokine secretion, reducing proliferation, and inducing apoptosis [100]. This interaction leads to the recruitment of the phosphatase SHP-2, which dephosphorylates components of the TCR signaling pathway, leading to the reduced activation and increased apoptosis of T cells [100]. These changes contribute to the disrupted immune homeostasis and prolonged immunosuppression observed in sepsis patients, ultimately exacerbating disease progression [91].

While these immune responses initially serve a protective function, their prolonged activation can disrupt immune homeostasis, leading to sepsis-induced immunosuppression. This sequence of regulatory responses and their consequences are effectively described by the “two-hit model” of sepsis, which illustrates how strong initial immune responses can lead to subsequent immunological vulnerabilities [101]. This model highlights the delicate balance required to manage the immune response during sepsis in order to prevent the transitioning from a protective to a harmful state.

The perspective on immunosuppression in sepsis shifted when it was revealed that many sepsis patients exhibit reduced immune activity, correlating with decreased survival rates. Specifically, a reduction in both naïve and memory CD8^+^ T cells, together with an increased expression of exhaustion markers like PD-1, compromises immune defenses and is associated with higher mortality rates [93,102,103]. The complexity of immunosuppression in sepsis is underscored by outcomes in clinical trials such as the CORTICUS trial [74]. In that trial, the use of glucocorticoids for the pan-inhibition of the immune response did not improve 28-day survival rates and actually increased the incidence of superinfections, including new cases of sepsis and septic shock [74]. These results highlight the risks associated with the over-suppression of the immune system and underscore the necessity of finely tuned immunomodulation strategies for sepsis management.

#### 2.2.2. Inhibition of Immune Paralysis as a Therapeutic Strategy for Sepsis

Strategies targeting immunosuppression were implemented after those targeting inflammation, meaning that fewer clinical trials have been conducted. Among the promising treatments, granulocyte–macrophage colony-stimulating factor (GM-CSF) and anti-PD-L1 antibodies are noteworthy (Table 1).

The interactions between PD-1 and PD-L1 have attracted considerable interest due to their critical role in promoting T-cell death and exhaustion, thereby exacerbating immune paralysis [92]. Extensive preclinical research has underscored the therapeutic potential of anti-PD-L1 antibodies in alleviating immunosuppression and bolstering immune function [93]. These studies have consistently shown positive results in murine models, suggesting that manipulating this pathway could improve clinical outcomes [104,105]. Additionally, this approach has been expanded to target other inhibitory molecules such as CTLA-4, which achieved similarly encouraging outcomes in diminishing the immunosuppressive environment [106].

However, not all strategies targeting inhibitory molecules have proven effective for treating sepsis. For example, in sepsis patients, elevated levels of T cell immunoglobulin and mucin domain protein 3 (Tim-3) are associated with increased mortality [92,107]. The use of soluble Tim-3 immunoglobulin (sTim-3-IgG) in murine models showed that blocking Tim-3 exacerbates early hyperinflammatory responses and lymphocyte apoptosis, and subsequently promotes a shift toward anti-inflammatory responses [108]. Furthermore, transitioning from the promising preclinical findings of PD-L1 inhibitors as a treatment for sepsis to definitive clinical outcomes remains a complex challenge. Recent initiatives such as the phase Ib trial by the Hotchkiss group focused on PD-L1 inhibition have not conclusively established the effectiveness of such interventions in human subjects [109,110,111].

GM-CSF has demonstrated promise in enhancing neutrophil phagocytic activities in critically ill patients with compromised immune responses, potentially reducing their risk of nosocomial infections [91]. A multicenter phase IIa study indicated that, while GM-CSF did not significantly increase the mean phagocytosis rates, it increased the proportion of patients achieving adequate phagocytosis levels [112]. In a phase II study, GM-CSF therapy significantly improved the Pa(O_2_)/FI(O_2_) ratio over 5 days (*p* = 0.02), indicative of enhanced gas exchange and pulmonary function, but did not increase the 30-day survival rates [113]. Additionally, a randomized, double-blind, placebo-controlled clinical trial found that GM-CSF reduced the duration of antibiotic treatment in patients with nontraumatic abdominal sepsis, but did not significantly affect the in-hospital mortality rates of these patients [114].

Treatment with IFN-γ has also been proposed as a strategy to correct immunosuppression in sepsis. IFN-γ, which enhances the macrophage phagocytic and bactericidal capabilities, significantly boosts monocyte HLA-DR expression and TNF-α secretion, aiding the eradication of pathogens [115]. However, no clinical trials have validated the efficacy of IFN-γ in the treatment of sepsis.

While the inhibition of immune paralysis using agents like GM-CSF and anti-PD-L1 antibodies shows substantial promise, the pathway from promising preclinical results to effective clinical applications remains fraught with challenges. Ongoing research and clinical trials are crucial for establishing reliable therapies to effectively combat sepsis-induced immunosuppression. To aid in understanding, we summarized the pro-inflammatory and anti-inflammatory cytokines discussed in this paper in Table 2.

### 2.3. Strategies for Mitigating Organ Damage in Sepsis

Targeting immune dysregulation addresses the causes of sepsis, while there is also research focused on strategies to manage the resulting organ dysfunction. After experiencing a cytokine storm, the target organs of sepsis patients suffer from internal endothelial disruption, metabolic derangement, and mitochondrial damage, leading to a loss of function [116,117]. This section will cover strategies that target specific organ cells to mitigate organ damage in sepsis.

**Figure 3:**
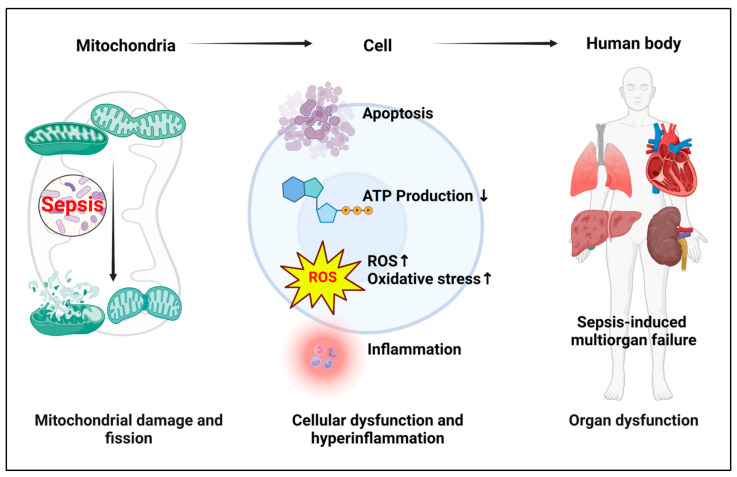
Impact of mitochondrial damage on sepsis-related organ failure. In sepsis, mitochondrial damage leads to increased fragmentation, which boosts apoptotic signaling and impairs the primary function of mitochondria, namely energy production. This failure results in heightened ROS production, exacerbating hyperinflammation. Ultimately, these disruptions contribute to cellular dysfunction and death, culminating in organ dysfunction.

Recent studies have increasingly focused on mitochondrial damage as a key mechanism in sepsis, particularly evident in various organs such as the kidneys, liver, and heart, where specific cell types—renal tubular epithelial cells, hepatocytes, and cardiomyocytes—are targeted [118,119,120,121,122,123]. The promotion of cell death signaling, increased oxidative stress, and disruptions in mitochondrial dynamics are some of the key factors contributing to mitochondrial dysfunction, which has been identified as a major cause of organ damage in sepsis [118]. One primary factor is that of oxidative stress, which leads to the overproduction of reactive oxygen species (ROS) that damage mitochondrial components, including lipids, proteins, and DNA, thereby impairing mitochondrial function and biogenesis [124]. This oxidative stress is exacerbated by the dysregulation of enzymes such as NADPH/NADH oxidase, cyclooxygenase, and xanthine oxidase, which further inhibit critical mitochondrial functions like the sarco/endoplasmic reticulum Ca^2+^ ATP-ase (SERCA) [125,126]. Additionally, mitochondrial Ca^2+^ overload disrupts the balance of mitochondrial dynamics, including fusion and fission processes, leading to further mitochondrial fragmentation and energy deficiency [125,126]. Thus, for example, Mdivi-1, a mitochondrial fission inhibitor, preserves mitochondrial integrity and attenuates cell death pathways during sepsis [127,128]. This approach offers a viable strategy for mitigating sepsis-induced organ damage by stabilizing mitochondrial dynamics, reducing ROS production, and lessening hyperinflammation (Figure 3).

Another example is in the context of sepsis-related acute kidney injury (AKI), where receptor-interacting protein kinase 3 (RIPK3) is significantly elevated and contributes to oxidative stress and mitochondrial dysfunction by upregulating NADPH oxidase-4 (NOX4) and inhibiting mitochondrial complexes I and III [122]. Additionally, RIPK3 exacerbates the kidney tubular injury by facilitating the mitochondrial translocation of NOX4 in response to proinflammatory stimuli, highlighting its role in the necroptotic pathway and inflammation in AKI [122]. Therapeutic strategies that aim to recover mitochondrial function have shown promising results in several preclinical studies for sepsis treatment. Although clinical trials are yet to commence, this remains a highly anticipated area of research.

## 3. Discussion

In this review, we explored the development of therapeutic agents aimed at addressing the pathogenesis of hyperinflammation and immune paralysis in sepsis, focusing on the progress of these treatments in clinical trials. Despite extensive efforts by pharmaceutical companies to modulate immune responses through methods such as blocking immune cell sensitization and employing monoclonal antibodies to reduce cytokine production, clinical trials have largely failed to improve the survival rates of sepsis patients. This disappointing outcome underscores the complexities involved in effectively treating sepsis and raises critical questions regarding the efficacy of current therapeutic strategies.

A primary reason why clinical trials in sepsis have failed stems from the inherent heterogeneity of the immune response among patients [129]. A clinical trial might focus on neutralizing a specific cytokine, such as TNF, based on the assumption that its upregulation contributes uniformly to disease progression in all patients [130]. However, this approach overlooks the broad range of cytokine responses, as illustrated by the finding that IL-6 concentrations vary from 8 to higher than 1.5 million pg/mL among sepsis patients [130,131]. Such variability indicates that targeting a single cytokine or pathway is unlikely to address the multifaceted and robust immune response, which is necessary to effectively tackle sepsis.

The variability in immune responses among sepsis patients also extends to the type of infecting microorganism, which adds complexity to the development of effective treatments. The type and nature of the pathogen can significantly influence the immune response, which current sepsis therapies should account for but often do not adequately address. For instance, while anti-TNF therapies might improve outcomes in cases involving endotoxins or specific bacteria like *Staphylococcus aureus*, they show limitations in more complex scenarios involving polymicrobial infections, as seen in models of sepsis induced by cecal ligation and puncture [132]. Additionally, the contrasting performance of IL-6 antagonists, which have shown limited effectiveness in bacterial infections but more success in COVID-19 clinical trials, further illustrates how the efficacy of treatments can vary according to the type of infection [59,133,134].

In addition to the heterogeneity in immune responses related to the type of infecting microorganism, there is also significant variability across different sites of infection and the related host responses. The immune response can vary greatly depending on whether the infection is localized to organs such as the lungs, urinary tract, or abdominal cavity [135]. This site-specific immune response adds another layer of complexity to developing effective sepsis treatments, as different sites of infection can trigger distinct immune pathways and outcomes.

Another reason why clinical trials in sepsis have failed is due to the complexities of immune dysregulation in septic patients with other preexisting immune-related diseases. Recent studies have highlighted the potential for better mortality outcomes in septic patients with certain autoimmune diseases compared to the general septic population. For example, patients with autoimmune conditions like systemic lupus erythematosus, multiple sclerosis, and rheumatoid arthritis tend to exhibit lower short-term mortality rates during sepsis hospitalizations [136,137,138]. It is hypothesized that pre-existing immune dysfunction in these patients alters their immune response to infection, leading to better outcomes. Analyzing the molecular differences in infection responses between these patients and the general population could enhance our understanding of the complexities of immune dysregulation in sepsis and its implications for future therapies.

In addressing the challenges of sepsis research, the diagnosis of sepsis itself poses a significant hurdle. Misclassification, where patients diagnosed with sepsis may in fact have different, noninfectious conditions, is a substantial issue. This includes scenarios where clinical diagnoses are confused with other serious conditions that mimic sepsis symptoms but are not caused by an infection, or where severe organ dysfunction occurs in the presence of an infection but is not directly attributable to a dysregulated host response. For instance, a case of acute hypoxic respiratory failure might be mistakenly attributed to sepsis when it is in fact caused by cardiogenic pulmonary edema from congestive heart failure exacerbated by a urinary tract infection. Such misdiagnoses can lead to the inclusion of inappropriate subjects in clinical trials, potentially obscuring the beneficial effects of treatments under study and contributing to the trials’ failures.

Moreover, the concept of a dysregulated host response to infection, a cornerstone of the Sepsis-3 framework, remains poorly defined. Despite the extensive descriptions of host response variations in sepsis, there is still no clear pathobiological explanation for the transition to a dysregulated response. Understanding these dynamics more clearly is crucial for advancing treatment strategies and improving patient outcomes in sepsis.

Another significant barrier to translating the findings of preclinical experiments into clinical success is the timing of intervention. Preclinical studies often benefit from the controlled and preemptive administration of immunosuppressive therapies. However, this level of control does not translate to clinical trials, where treatments typically begin only after sepsis is formally diagnosed. The diagnosis is based on the “Sepsis-3” consensus definitions and the sepsis-related organ failure assessment (SOFA) score, which evaluates criteria including the PaO_2_/FiO_2_ ratio, Glasgow Coma Scale score, mean arterial pressure, administration of vasopressors, serum creatinine level, urine output, and bilirubin level [1]. The manifestation of these criteria signifies that significant organ dysfunction has already occurred. Despite ongoing research efforts, the precise mechanisms of organ dysfunction in sepsis remain incompletely understood, and the concept of a cytokine storm is not uniformly applicable to all cases of sepsis. For example, in COVID-19-related sepsis, the role of cytokines is still being studied and understood. This gap in our understanding of sepsis pathobiology is partly responsible for the failure of many clinical studies.

In addition to the challenges of timing interventions in clinical trials, the diversity of patient characteristics presents another significant hurdle. This diversity is not just confined to the reaction of the immune system but extends to the genetic makeup and comorbid conditions of patients [131,139]. The transition from the controlled environment of preclinical studies to the unpredictable and varied conditions of clinical settings introduces numerous variables that can impact the efficacy of a therapy. Patients in clinical trials have a wide range of genetic backgrounds, underlying conditions, and stages of disease progression, all of which can influence the outcome of the treatment.

Additionally, the immune response in humans, particularly in patients with sepsis, is more complex and can significantly differ from that in animal models. There are substantial cross-species differences in response to infection or lipopolysaccharide (LPS) in sepsis models. For instance, the timing and dosage of LPS administration, the type of insult, and specific interventions can vary significantly between animal models and human sepsis, as sepsis in animal models is often induced rapidly with high doses of LPS or by the direct introduction of bacteria, which may not accurately replicate the progression of sepsis in humans [140]. Moreover, the progression and timeline of sepsis can be markedly different, with human sepsis often developing over days to weeks, whereas animal models typically show a more acute course. Furthermore, factors such as glucose levels and temperature, which are managed in preclinical sepsis models, can vary widely in human patients and affect the course of the disease and its response to treatment [141]. These complexities underscore the difficulties of applying findings from preclinical studies directly to clinical practice and highlight the need for innovative approaches to bridge this gap. Both the design and implementation of clinical trials must be reevaluated to ensure that they more accurately reflect the heterogenous nature of human sepsis.

As the limitations of traditional, uniform approaches in sepsis therapy become apparent, there is a growing emphasis on personalized therapy [142,143]. This approach underscores the potential for adapting treatments to meet individual patient needs, demonstrating the critical importance of personalized interventions in sepsis management. The efficacy of glucocorticoids, as noted in the results section, varies across clinical trials. However, glucocorticoids consistently yield positive outcomes in patients with heightened immune responses, especially when cytokine levels are elevated. Additionally, corticosteroids effectively reduce the duration of vasopressor infusion in patients requiring vasopressor support, showcasing their capability to manage septic shock under specific inflammatory conditions [144]. A meta-analysis further confirmed that corticosteroids enhance outcomes in sepsis, especially when cytokine levels are elevated [145]. The effectiveness of TNF-α inhibitors for improving treatment outcomes in patients with high IL-6 levels further underscores the value of personalized medicine [45]. These findings emphasize the importance of personalized therapy, suggesting that categorizing patients based on their immune status can lead to more predictable therapeutic responses.

Recent advancements in sepsis management have highlighted the importance of profiling and phenotyping approaches, which enable the development of personalized therapies specifically designed for individual patient characteristics. Considering the heterogeneity of the disease, these methods have become increasingly vital, as they allow for the more precise targeting of treatments that can significantly enhance both the precision of care and patient outcomes.

Cytokine profiling is a fundamental tool in the personalized management of sepsis, enabling the precise customization of therapies based on distinct cytokine profiles to effectively meet individual patient needs [146]. The study by Matsumoto et al. demonstrates the strong association of cytokines like IL-6, IL-8, MCP-1, and IL-10 with disease severity and prognosis, evidenced by their significant correlations with SOFA scores and markers of disseminated intravascular coagulation [37]. Furthermore, multiplex cytokine profiling has been employed to link specific cytokine levels with varying degrees of sepsis severity, where cytokines serve not only as prognosis markers but also as targets for therapeutic intervention [147,148]. For example, IL-8 and MCP-1 are strongly correlated with organ dysfunction and mortality, proving useful as early markers for risk stratification [147]. Additionally, cytokine levels such as IL-6, IL-8, and G-CSF within the first 24 h can predict worsening organ dysfunction, aiding clinicians in monitoring disease progression and adjusting treatments accordingly [148].

Research on sepsis phenotyping extends to areas such as transcriptomics and broader immunophenotyping, which encompass not only cytokines but also various protein biomarkers and cell surface markers. Techniques such as high-dimensional flow cytometry and transcriptomic analyses delineate specific immune phenotypes, such as hypo-responsiveness and hyper-responsiveness. These phenotypes correlate strongly with clinical outcomes and can guide the application of specialized treatments, reducing the one-size-fits-all approach, and increasing the likelihood of positive outcomes [149]. This approach was illustrated in the PROVIDE randomized clinical trial, which highlighted the importance of personalized therapy in managing immune paralysis, identified through notably low HLA-DR expression on monocytes (less than 5000 receptors per monocyte). Patients with this phenotype showed a distinct response profile to treatment interventions, which underscores the value of specifically customizing therapies to such profound immunosuppression [150]. However, it is important to acknowledge the limitations of these phenotypes, especially those generated by post hoc analyses. Such analyses often suffer from imbalances in patient characteristics, unlike the controlled settings of whole-cohort randomizations in randomized clinical trials. This imbalance can skew the accuracy and applicability of the phenotypes derived, potentially limiting their utility in clinical practice.

The integration of artificial intelligence (AI) and machine learning into sepsis management has significantly advanced personalized medicine, enhancing the precision of treatments. For example, the application of the deep deterministic policy gradient (DDPG) algorithm in AI-based medical decision-making systems has been shown to reduce patient mortality rates by providing optimal dosing combinations that closely align with those recommended by professional clinicians [151]. Additionally, the development of real-time, personalized sepsis prediction frameworks combining electrocardiogram data with electronic medical records enables accurate prediction of sepsis onset up to four hours before clinical symptoms appear, making them suitable for at-home monitoring and reducing the need for invasive laboratory tests [152]. Despite these advancements, challenges remain in the implementation and generalization of these models, including the need for data enrichment from sources beyond the electronic health record and the necessity for rigorous prospective studies to validate these algorithms in clinical settings [151].

These findings underscore the necessity of phenotypic categorization and personalized therapeutic approaches, enabling clinicians to better predict patient responses to treatment and ultimately improve outcomes.

## 4. Conclusions

The frequent failures of sepsis therapies point to the need for a paradigm shift toward more personalized and timely interventions. As we continue to dissect the complex cytokine network and varied immune responses in sepsis patients, future research should prioritize phenotype-driven, personalized approaches that consider the unique profiles of individual patients to enhance treatment outcomes in sepsis management.

## Figures and Tables

**Table 1:** Characteristics of immune dysregulation in sepsis.

	Hyperinflammation	Immunosuppression
Factors	Pro-inflammatory cytokines (TNF-α, IL-6, and IL-1β) DAMPs (HMGB1, HSPs, DNA, and RNA) NETosis Complement activation	Anti-inflammatory cytokines (IL-4, IL-10, IL-37, and TGF-β) Upregulation of negative costimulatory molecules (PD1, PD-L1, CTLA4, and TIM3) Proliferation of immunosuppressor cells (MDSCs and FoxP3^+^ T regulatory cells)
Main cause of death ^1^	Cytokine storm-induced organ dysfunction	Weakened clearance of infection Activation of secondary infection
Clinical trial	TLR inhibitors (Eritoran and TAK-242) AP CytoSorb Polymyxin B Anti-TNF-α monoclonal antibody IL-1β receptor inhibitor IL-6 antagonist IVIg Glucocorticoids	PD-L1 inhibitor GM-CSF

^1^ Disease-aggravating factors.

**Table 2:** Summary of the role of pro- and anti-inflammatory cytokines in sepsis.

Cytokine	Role in Sepsis	References
TNF-α	Pro-inflammatory cytokine, produced shortly after infection onset	[37,38,39]
IL-1β	Pro-inflammatory cytokine, triggers release of other cytokines	[37,48]
IL-6	Plays a dual role by initiating both pro- and anti-inflammatory reactions, presents in prolonged period	[37,62,63]
IL-4	Anti-inflammatory cytokine, upregulated to mitigate inflammation and promote tissue recovery	[94]
IL-10	Anti-inflammatory cytokine, suppresses T cell responses, inhibits effector T cell function	[95]
IL-37	Anti-inflammatory cytokine, upregulated to mitigate inflammation and promote tissue recovery	[96]
TGF-β	Anti-inflammatory cytokine, suppresses effector T cell function, but has detrimental effects on disease outcomes by promoting fibrosis	[97]
